# Photobiomodulation promotes repair following spinal cord injury by restoring neuronal mitochondrial bioenergetics *via* AMPK/PGC-1α/TFAM pathway

**DOI:** 10.3389/fphar.2022.991421

**Published:** 2022-09-12

**Authors:** Zhijie Zhu, Xuankang Wang, Zhiwen Song, Xiaoshuang Zuo, Yangguang Ma, Zhihao Zhang, Cheng Ju, Zhuowen Liang, Kun Li, Xueyu Hu, Zhe Wang

**Affiliations:** Department of Orthopedics, Xijing Hospital, Fourth Military Medical University, Xi’an, China

**Keywords:** spinal cord injury, photobiomodulation, mitochondria, AMPK, PGC-1α, TFAM, neuron

## Abstract

**Background:** Insufficient neuronal mitochondrial bioenergetics supply occurs after spinal cord injury (SCI), leading to neuronal apoptosis and impaired motor function. Previous reports have shown that photobiomodulation (PBM) could reduce neuronal apoptosis and promote functional recovery, but the underlying mechanism remains unclear. Therefore, we aimed to investigate whether PBM improved prognosis by promoting neuronal mitochondrial bioenergetics after SCI.

**Methods:** Sprague Dawley rats were randomly divided into four groups: a Sham group, an SCI group, an SCI + PBM group and an SCI + PBM + Compound C group. After SCI model was established, PBM and Compound C (an AMPK inhibitor) injection were carried out. The level of neuron apoptosis, the recovery of motor function and mitochondrial function were observed at different times (7, 14, and 28 days). The AMPK/PGC-1α/TFAM pathway was hypothesized to be a potential target through which PBM could affect neuronal mitochondrial bioenergetics. *In vitro*, ventral spinal cord 4.1 (VSC4.1) cells were irradiated with PBM and cotreated with Compound C after oxygen and glucose deprivation (OGD).

**Results:** PBM promoted the recovery of mitochondrial respiratory chain complex activity, increased ATP production, alleviated neuronal apoptosis and reversed motor dysfunction after SCI. The activation of the AMPK/PGC-1α/TFAM pathway after SCI were facilitated by PBM but inhibited by Compound C. Equally important, PBM could inhibit OGD-induced VSC4.1 cell apoptosis by increasing ATP production whereas these changes could be abolished by Compound C.

**Conclusion:** PBM activated AMPK/PGC-1α/TFAM pathway to restore mitochondrial bioenergetics and exerted neuroprotective effects after SCI.

## Introduction

Spinal cord injury (SCI) is mainly caused by violence factors and there remains no effective treatment for motor function disorder. Mitochondria carry out aerobic respiration and are a source of bioenergy that their dysfunction is considered to be a key factor in the aggravation of injury and subsequent neuronal cell death after SCI (Rabchevsky et al., 2020). When primary injury causes demyelination and axonal degeneration, it could also directly cause severe damage to mitochondria, resulting in bioenergetics metabolism disorder, and aggravating demyelination (Rogers and Todd, 2016; Slater et al., 2022). During secondary injury, the oxidative respiratory chain is broken and an insufficient bioenergetics supply contributes to irreversible damage to axons and nerve cell bodies (Beattie et al., 2002; Scholpa and Schnellmann, 2017). Recently, increasing attention has been paid to strategies targeting mitochondrial bioenergetics to treat SCI.

Photobiomodulation (PBM), also called low-level laser therapy, has been widely used in the treatment of burns, stroke, SCI, peripheral nervous system injury and central nervous system degenerative diseases (Gigo-Benato et al., 2005; Naeser et al., 2014; Hamblin, 2017). PBM activates cytochrome c oxidase, enhances mitochondrial function, and ultimately promotes mitochondrial bioenergy, namely adenosine triphosphate (ATP) production (Chung et al., 2012). Previous studies have largely focused on increased ATP production rather than the specific molecules or pathways involved (Chang et al., 2019; Negri et al., 2019; Chaudary et al., 2020). Recently, Ravera et al. found that both 980 and 1,064 nm laser exposure could improve the activity of some mitochondrial complexes and thereby increase ATP production (Ravera et al., 2019; Amaroli et al., 2021). However, the specific signaling pathway through which PBM regulates mitochondrial bioenergetics remains unclear.

Mitochondrial bioenergetics refers to mitochondrial repair, growth, and fusion, and it involves a complex network of pathways regulating a variety of mitochondrial DNA coding genes. Adenosine 5'-monophosphate (AMP)-activated protein kinase (AMPK) is the most important regulator of cellular bioenergetics. When mitochondria are damaged, AMPK phosphorylates downstream enzymes that activate signaling cascades to increase ATP production and reduce ATP consumption, thereby restoring energy balance (Herzig and Shaw, 2018). Peroxisome proliferative activated receptor γ (PPARγ) coactivator 1α (PGC-1α) is another regulator of mitochondrial bioenergetics that interacts with several transcription factors (Fu et al., 2016). After SCI, the levels of PGC-1α and some electron transport chain subunits decrease, while LY344864 upregulates these proteins to pre-injury levels (Simmons et al., 2020). Mitochondrial transcription factor A (TFAM) not only promotes mitochondrial DNA transcription but also participates in the regulation of mitochondrial complex synthesis. Mitochondrial respiratory chain complex enzyme activity decreases in mice with myocardial infarction, and this phenomenon was revered in TFAM transgenic mice (Ikeuchi et al., 2005). Similar results have been reported in studies of memory impairment (Hayashi et al., 2008). In addition, transcription factors such as nuclear respiratory factor 1 (Nrf1) and sirtuin-1 (Sirt1) are also involved in the regulation of the AMPK-PGC-1α-TFAM axis and, in myocardial infarction and diabetic peripheral neuropathy, a relationship between AMPK, PGC-1α, Sirt1, Nrf1, and TFAM was further established through the use of corresponding inhibitors and activators (Tian et al., 2019; Zhang et al., 2021).

Of note, Zhang et al. established that PGC-1α and TFAM expression were upregulated and ATP production was increased after PBM intervention in hematological disorders (Zhang et al., 2016). We hypothesized that this phenomenon will also occur after SCI. Therefore, this study aimed to determine whether PBM could increase ATP production by regulating the AMPK/PGC-1α/TFAM pathway and thereby reducing mitochondrial-related apoptosis and improving SCI prognosis. Using an *in vivo* SCI model, along with oxygen-glucose deprivation (OGD) model of ventral spinal cord 4.1 (VSC 4.1) motoneuron cells *in vitro*, our results suggested that PBM could promote mitochondrial bioenergetics *via* the AMPK/PGC-1α/TFAM signaling pathway.

## Materials and methods

### Animal model, photobiomodulation therapy, and drug administration

All animal experiments were approved by the Institutional Animal Care and Use Committee of the Air Force Medical University. Male Sprague Dawley rats were purchased from the Animal Center of the Air Force Medical University. SCI model, laser fiber embedding and photobiomodulation therapy were the same as our previous research (Wang et al., 2021a). After the rats were anesthetized with pentobarbital sodium, the lamina was excised, the T10 spinal cord was exposed and clamped with special pliers for 40 s to cause SCI. The Sham group only opened the lamina. In SCI + PBM group, biocompatible laser fibers (MW-GX-808, Lei Shi Optoelectronics Co., Ltd. Changchun, China) were embedded into the lamina of T8 and T12 so that the T10 spinal cord could be exposed to PBM an hour every day. More details about the treatment are presented in Supplementary Table S1. Routine animal care was performed after operation. Drug administration were conducted as previously described (Jiang et al., 2020). Compound C (CC) (HY-13418a, MCE, United States) was dissolved in DMSO and normal saline successively. SCI + PBM + CC group was injected with Compound C intraperitoneally (10 mg/kg) once a day from 7 to 14 dpi.

### Gait analysis

The dorsum of the hindlimbs was smeared with red ink pad, and the soles of the hindlimbs were smeared with blue ink pad. Animals were placed on the aisle covered with white paper (50 cm long × 20 cm wide) to walk forward. The distance between two adjacent steps on the same side is called stride length (Li et al., 2019). Each animal was measured repeatedly and the average value was obtained as the final result to reduce accidental error.

### Slice preparation

Animals were perfused with paraformaldehyde at specific time points, and a segment of about 2 cm was excised from the injured spinal cord, immersed in 4% paraformaldehyde fixative for 24 h, and placed in 25% sucrose solution overnight. The tissue was embedded with OCT, and the sectioning were started after the embedding medium was completely solidified. Approximately 7 μm thick serial sagittal sections were obtained and reserved.

### Immunofluorescence

Sections was treated with 0.3% TritonX-100 for 20 min and blocked with 5% BSA blocking solution for 1 h at room temperature. Corresponding primary antibody was added to the sections and incubated overnight at 4°C. Sections was incubated with secondary antibody (1:200) at room temperature for 1 h in the dark. Nuclei were stained with DAPI after washing with PBS for 10 min. The antibodies used are as follows: anti-NeuN (1:400, ab104224, Abcam, United Kingdom), anti-MAP2 (1:400,8707T, Cell Signaling Technology, United States), anti-TFAM (1:400, ab252432, Abcam, United Kingdom), anti-PGC-1α (1:100,66369-1-Ig, Proteintech, China), TOM20 (1:400, 11802-1-AP, Proteintech, China), Mito Tracker red CMXRos (100 nM, M7512, Invitrogen, United States). Cells were fixed with paraformaldehyde for 30 min before staining, and other progresses remained unchanged. The fluorescence intensity was calculated by ImageJ.

### TUNEL assay

The TUNEL working detection solution was prepared by using the TUNEL detection kit (C1090, Beyotime, China), mixed with the corresponding secondary antibody, and incubated with the slice at room temperature for 1 h. The proportion of TUNEL-positive neurons was counted by ImageJ.

### Image analysis and quantification

Pictures were taken randomly among the area of 600 μm rostral and caudal to the lesion area. Fluorescence intensity was analyzed after calibration with an empirical method (Karimi-Abdolrezaee et al., 2006). When calculating the ratio of TUNEL positive neurons, try to keep the number of neurons in each group the same to reduce the accidental error. All analytical quantifications were done by two investigators who were blinded to the experiment.

### Western blotting

Animals were perfused with normal saline at a specific time point, and about 2 cm segments were cut from the injured spinal cord. Then, protein was isolated by homogenizing the segments after RIPA lysis. The protein concentration was obtained by the BCA assay. After electrophoresis, membrane transfer, blocking, the membrane was incubated with primary antibody overnight at 4°C. The following antibodies were used: anti-AMPK(1:2000,10929-2-AP, Proteintech, China), p-AMPK(1:2000, 2535T, Cell Signaling Technology, United States), anti-PGC-1α(1:1000, 66369-1-Ig, Proteintech, China),anti-TFAM(1:2000, ab252432, Abcam, United Kingdom), anti-Nrf1(1:2000, ab175932, Abcam, United Kingdom),anti-Sirt1(1:1000, 13161-1-AP, Proteintech, China),Anti-NDUFB8(Complex I) (1:2000, ab192878, Abcam, United Kingdom), Anti-SDHB(Complex Ⅱ) (1:2000, ab175225, Abcam, United Kingdom), Anti-UQCRC2 (Complex Ⅲ) (1:2000, abs116449, absin, China), Anti-COX IV (Complex IV) (1:2000, 4844s, Cell Signaling Technology, United States), Anti- ATP5F1A (Complex V) (1:2000, ab176569, Abcam, United Kingdom), anti-Beta Actin (1:5000, 66009-1-Ig,Proteintech, China). Membrane was incubated with the corresponding secondary antibody at room temperature for 1 h. Using ECL hypersensitive luminous solution to visualize the imprint and analyze the fluorescence intensity.

### Transmission electron microscope

Sample was prefixed 3% glutaraldehyde, refixed with 1% osmium tetroxide and dehydrated with acetone. Ep812 embedded the sample before it was sliced. Uranium acetate and lead citrate stained the sample and then it was observed by JEM-1400FLASH transmission electron microscope.

### Adenosine triphosphate assay

Enhanced ATP assay kit (S0027, Beyotime, China) was used to detected ATP levels of tissues and cells. The sample was added into the lysis solution and then homogenized, centrifuged at 4°C 12,000 g for 5 min, and took the supernatant for subsequent determination.

### Oxygen and glucose deprivation model and processing

The ventral spinal cord 4.1 (VSC4.1) motor neuron cells were cultured in DMEM sugar-containing medium containing 10% fetal bovine serum and 100 U/m penicillin and streptomycin in a 37°C incubator. Before starting OGD treatment, the medium was aspirated and washed with PBS. Sugar-free DMEM medium (11966025, Thermo Fisher, United States) was added to each well, and an equal volume of sugar-containing culture medium was added to the control group. The well plate was placed in a hypoxia bag (BioMèrieux, Marcy I 'etoile, France) for 8 h (Li et al., 2019). Then the sugar-free medium was removed and replaced with the original medium, and the cells were cultured in an incubator with normal parameters (reoxygenation, 37°C, 5% CO_2_) for 24 h before the next procession. The OGD + PBM group received two times of PBM for 10 min within 24 h at fixed time (9 and 21 a.m.) (Wang et al., 2021b), and Compound C (10 µM, 24 h) was added to the OGD + PBM + CC group to inhibit the AMPK pathway. In order to exclude the toxic effect of DMSO on cells, equal volume of DMSO was added to the OGD + DMSO group. After the above processes (56 h) were completed, Western blot and immunofluorescence staining was performed.

### Statistical analysis

The GraphPad prism eight software was used for statistical analysis. Student’s *t*-test was used for the comparison of the two groups. One way ANOVA was used to compare the differences between groups at specific time points, two-way ANOVA was used to compare the differences between groups at different time points, and Bonferroni was used as a post test. The statistical difference was defined as *p* < 0.05, and the data were expressed as mean ± standard deviation (x ± SD). *p < 0.05, **p < 0.01, ***p < 0.001, NS, no significant.

## Results

### Photobiomodulation promoted motor function recovery and alleviated mitochondrial-related neuronal apoptosis after spinal cord injury

**FIGURE 1:**
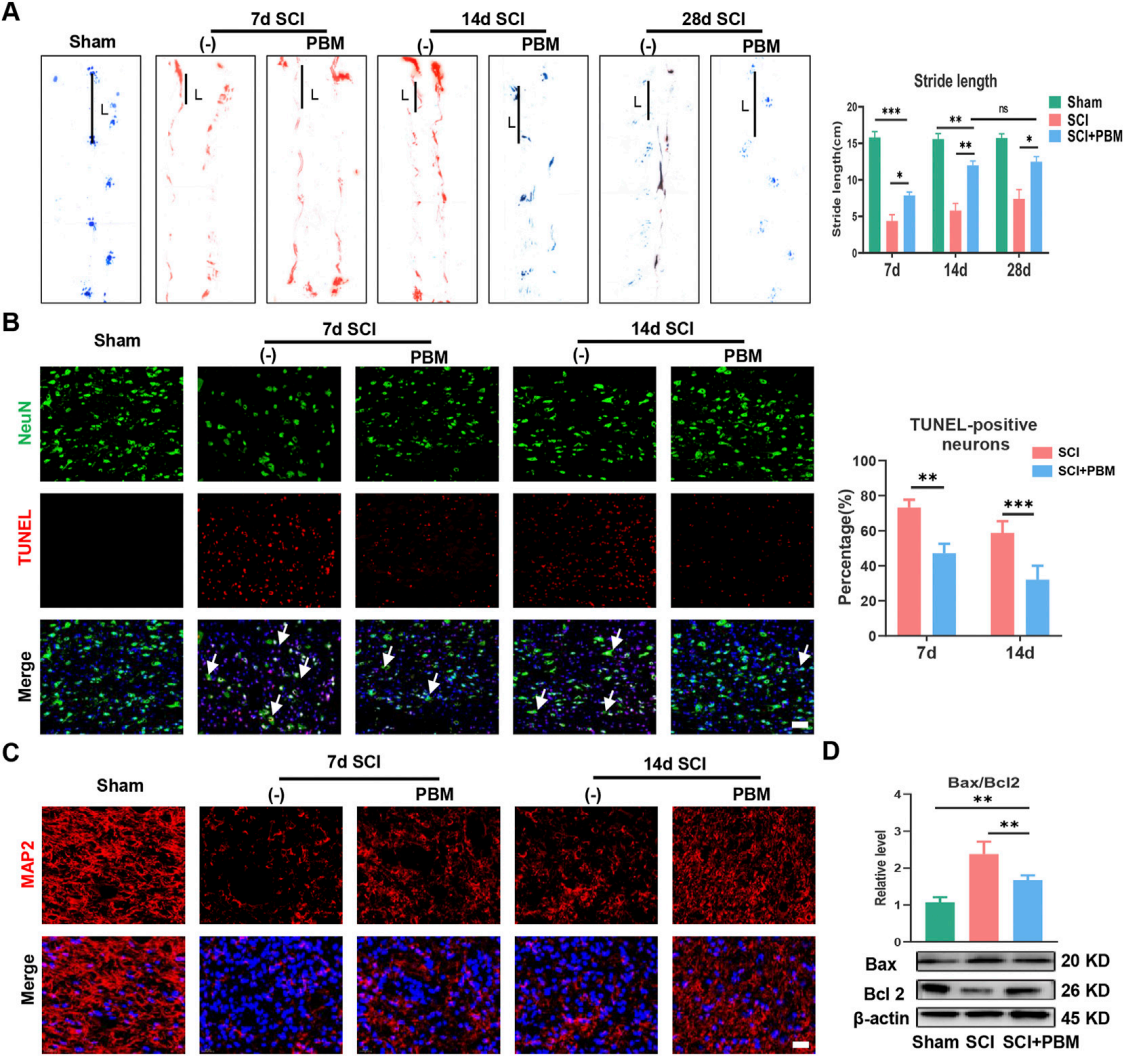
PBM promoted motor function recovery and alleviated mitochondrial-related neuronal apoptosis. **(A)**: Representative gaits of each group at 7, 14, and 28 dpi. The stride length was calculated, *n* = 3. **(B)**: TUNEL (red) and NeuN (green) co-stained and the arrows point to representative cells. The ratio of TUNLE-positive neurons was calculated, Scale bar: 50 µm, *n* = 3. **(C)**: Neurons was stained with MAP2, and nuclei were stained with DAPI (blue), Scale bar: 50 µm. **(D)**: Western blot analysis and quantification of Bax/Bcl2 levels in each group, *n* = 4.

We first assessed the effect of PBM on motor function recovery *via* behavioral analysis. Gait analysis showed that rat hindlimbs clearly stepped on the ground with a stable stride; the longest stride length was observed in the Sham group (Figure 1A). In contrast, the SCI group had a trailing gait with a shorter stride. In addition, hindlimb stride length in the SCI + PBM group was longer than that in the SCI group from 7 to 28 days (Figure 1A, SCI + PBM group vs. SCI group: 7.85 ± 0.49 vs. 4.36 ± 0.86, 7 dpi; 11.96 ± 0.60 vs. 5.80 ± 0.97, 14 dpi; 12.466 ± 0.70 vs. 7.40 ± 1.27,28 dpi). Interestingly, there was no significant difference in stride length between 14 and 28 days after PBM intervention. This suggested that the ideal therapeutic time window for PBM is 14 days, consistent with the results of previous meta-analysis (Ayar et al., 2021). Next, we decided to more closely examine neuronal apoptosis at 7 and 14 days since there was no notable difference in motor function between 14 and 28 days after PBM intervention. A large number of apoptotic neurons appeared after SCI and PBM significantly reduced the percentage of TUNEL-positive neurons (Figure 1B, SCI + PBM group vs. SCI group: 47.11 ± 5.40 vs. 73.28 ± 4.40, 7 dpi; 32.07 ± 7.95 vs. 58.74 ± 6.69, 14 dpi). MAP2 labels neuronal cell bodies and axons and we found that PBM could promote expression of MAP2 at both 7 and 14 dpi (Figure 1C), consistent with the trend of PBM inhibition of neuronal apoptosis (Figure 1B). Third, we investigated whether neuronal apoptosis was associated with mitochondrial damage. Bax, which promotes mitochondrial membrane permeability and leads to apoptosis, increased after SCI, while Bcl-2, which inhibits enhancement of mitochondrial membrane permeability, decreased after SCI. This trend was reversed after PBM intervention (Figure 1D).

### Photobiomodulation activated the AMPK/PGC-1α/TFAM pathway after spinal cord injury

**FIGURE 2:**
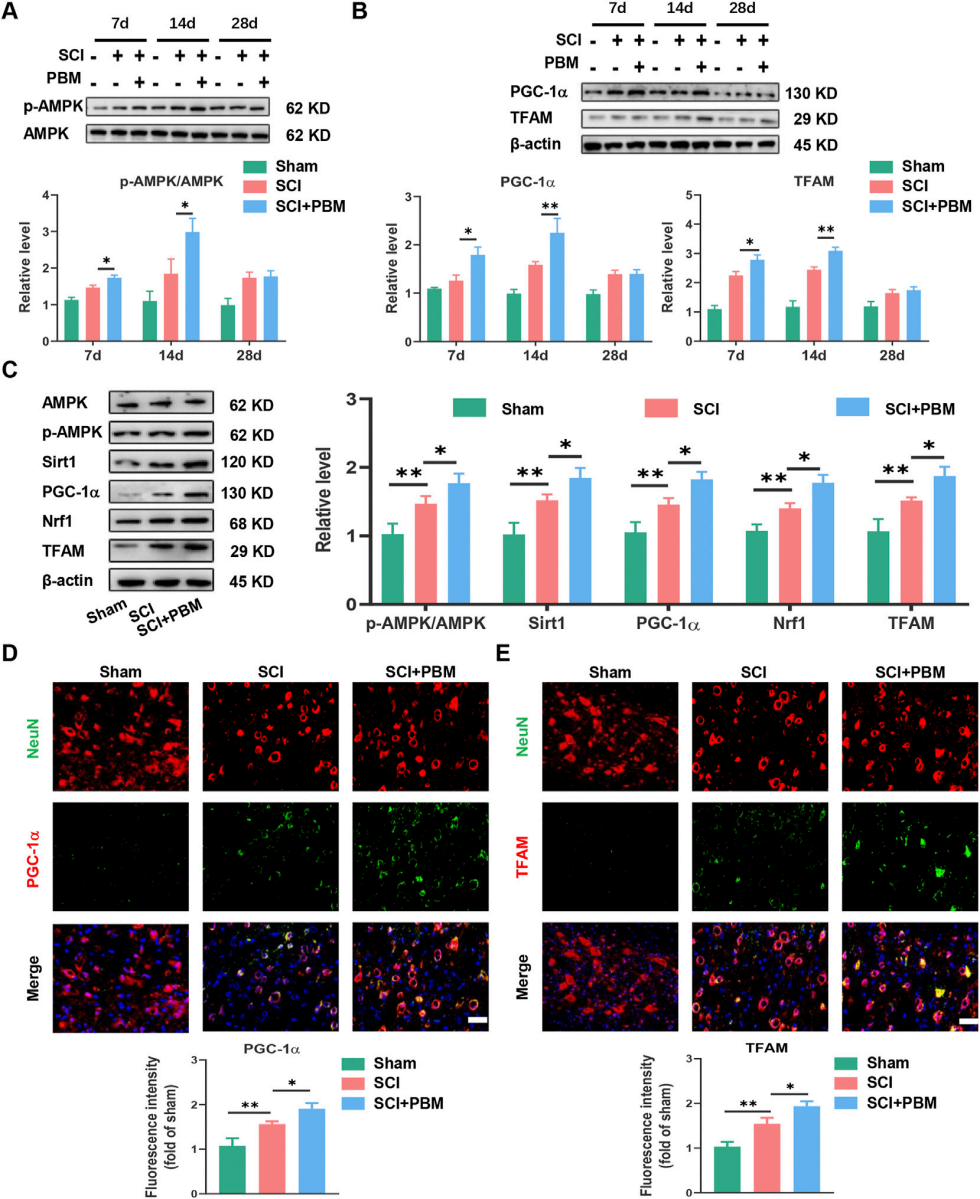
PBM activated the AMPK/PGC-1α/TFAM pathway after SCI. **(A)**: Western blot analysis and quantification of p-AMPK/AMPK expression levels at 7, 14, and 28 dpi in each group, *n* = 3. **(B)**: Western blot analysis and quantification of PGC-1α, TFAM expression levels at 7, 14, and 28 dpi in each group, *n* = 3. **(C)**: Western blot analysis and quantification of p-AMPK/AMPK, PGC-1α,TFAM, Sirt1, and Nrf1 expression levels in each group at 14 dpi, *n* = 3. **(D)**: Representative images of NeuN (red) and PGC-1α (green) for each group at 14 dpi. Nuclei were stained with DAPI (blue). Quantification of the intensity of PGC-1α relative to that in the sham group, Scale bar: 50 µm, *n* = 3. **(E)**: Representative images of NeuN (red) and TFAM (green) for each group at 14 dpi. Nuclei were stained with DAPI (blue). Quantification of the intensity of TFAM relative to that in the sham group, Scale bar: 50 µm *n* = 3.

Western blot (WB) analysis showed that, compared to the SCI group, phosphorylated AMPK (p-AMPK) increased significantly at 7 days, peaked at 14 days, and then decreased gradually (Figure 2A). In addition, expression of TFAM and PGC-1α (Figure 2B) showed a dynamic trend similar to p-AMPK (Figure 2A). The dynamic trend of these molecules was aligned with the timeline of motor function recovery and neuronal apoptosis reduction after PBM intervention mentioned above (Figure 1). Therefore, we targeted day 14 for follow-up experiments. We found that, compared to the SCI group, expression levels of p-AMPK/AMPK, PGC-1α, TFAM, Sirt1, and Nrf1 were significantly increased in the SCI + PBM group (Figure 2C). Furthermore, in line with our WB results, similar changes in TFAM and PGC-1α in neurons of three groups were confirmed by immunofluorescence (Figures 2D,E).

### Photobiomodulation promoted an increase in mitochondrial bioenergetics at 14 dpi

**FIGURE 3:**
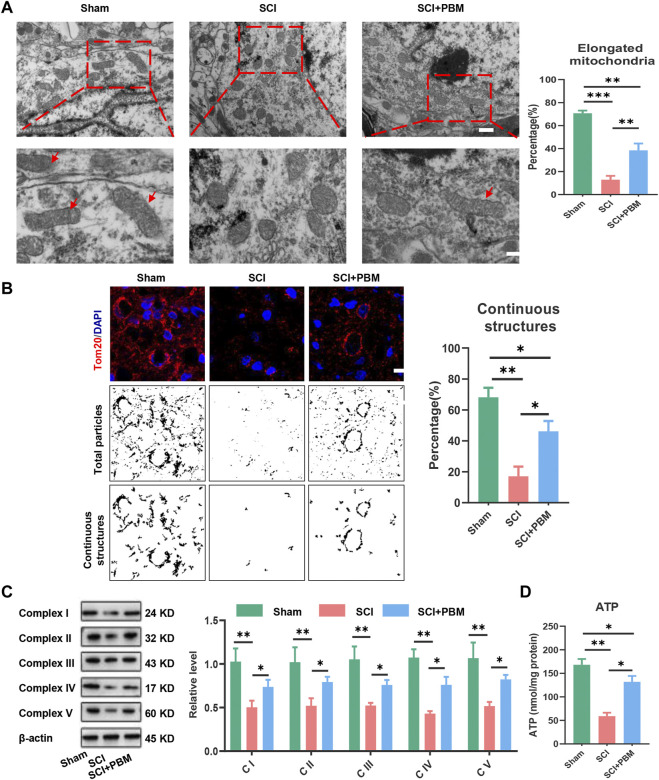
PBM promoted an increase in mitochondrial bioenergetics at 14 dpi. **(A)**: Representative images of neuronal mitochondria in each group by TEM. Qualitative analysis of the proportion of elongated (>2 μm in size) mitochondria. Representative mitochondria are indicated by red arrows. Upper panel scale bar: 2 μm. Lower panel scale bar: 1 μm *n* = 5. **(B)**: Representative confocal images of mitochondria stained with Tom20 (red) at 14 dpi. Nuclei were stained with DAPI (blue). Images were separated, thresholded, filtered and binarized with ImageJ. Continuous mitochondrial structures were calculated as the percentage of the area of large particles normalized to the total mitochondrial particles area, Scale bar: 10 µm, *n* = 5. **(C)**: Western blot analysis and quantification of the expression levels of Complex I–V in each group, *n* = 3. **(D)**: Result of ATP levels in each group, *n* = 4.

At the morphological level, transmission electron microscopy (TEM) and immunofluorescence (IF) were used to evaluate the ratio of functional mitochondria that are available to produce ATP. TEM results showed that mitochondria were swollen and fragmented after SCI, and the ratio of functional mitochondria (longer than 2 μm) (Gomes et al., 2011; Chalmers et al., 2016) decreased while PBM alleviated mitochondrial fragmentation (Figure 3A). Tom20 (Figure 3B) was used to label mitochondria and analyze continuous structures (size >2 μm). These structures were considered as functional mitochondria (Lu et al., 2017), and the results were similar to those of TEM. Next, we investigated mitochondrial functional changes at the molecular level. Mitochondrial respiratory complex proteins mediate the process of oxidative phosphorylation and ATP production. Our results showed that downregulation of Complex I–V expression could be recovered by PBM intervention (Figure 3C). Finally, detection of ATP indicated that PBM could increase ATP production after SCI (Figure 3D).

### Inhibition of AMPK/PGC-1α/TFAM pathway reversed the effects of Photobiomodulation *in vivo*

**FIGURE 4:**
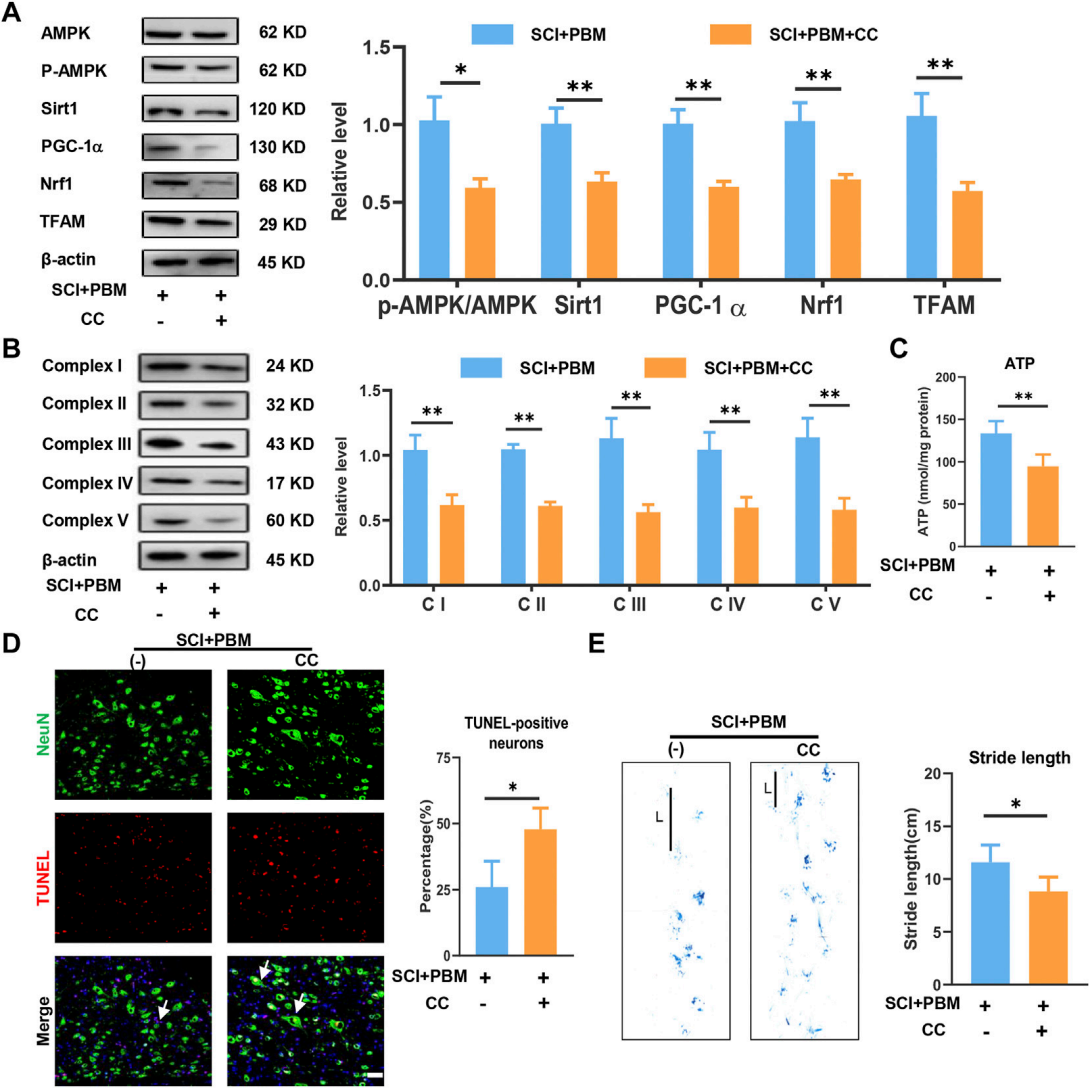
Inhibition of AMPK/PGC-1α/TFAM pathway reverses the effects of PBM *in vivo*. **(A)**: Western blot analysis and quantification of p-AMPK/AMPK, PGC-1α,TFAM, Sirt1, and Nrf1expression levels at 14 dpi in each group, *n* = 3. **(B)**: Western blot analysis and quantification of the expression levels of Complex I–V at 14 dpi in each group, *n* = 3. **(C)**: Result of ATP levels at 14 dpi in each group, *n* = 4. **(D)**: TUNEL (red) and NeuN (green) co-stained and arrows point to representative cells. The ratio of TUNLE-positive neurons was calculated, Scale bar: 50 µm, *n* = 4. **(E)**: Gait analysis and representative gaits at 14 dpi of each group. The stride length was calculated, *n* = 4.

The results described above suggest possible effects of PBM on AMPK/PGC-1α/TFAM pathway activation, mitochondrial bioenergetics, neuronal apoptosis, and motor function recovery. Compound C (CC), an AMPK inhibitor, was co-applied with PBM to assess whether the AMPK pathway was a potential target through which PBM could affect the outcomes after SCI. We found that levels of p-AMPK/AMPK, PGC-1α, TFAM, Sirt1 and Nrf1 were lower in the SCI + PBM + CC group compared to the SCI + PBM group (Figure 4A). In addition, complex I–V levels were lower (Figure 4B) and ATP production was decreased (Figure 4C) in the SCI + PBM + CC group. Finally, compared to the SCI + PBM group, the SCI + PBM + CC group showed a higher level of neuronal apoptosis (Figure 4D) and a shorter stride length in gait analysis (Figure 4E).

### Photobiomodulation activated AMPK/PGC-1α/TFAM pathway *in vitro*

**FIGURE 5:**
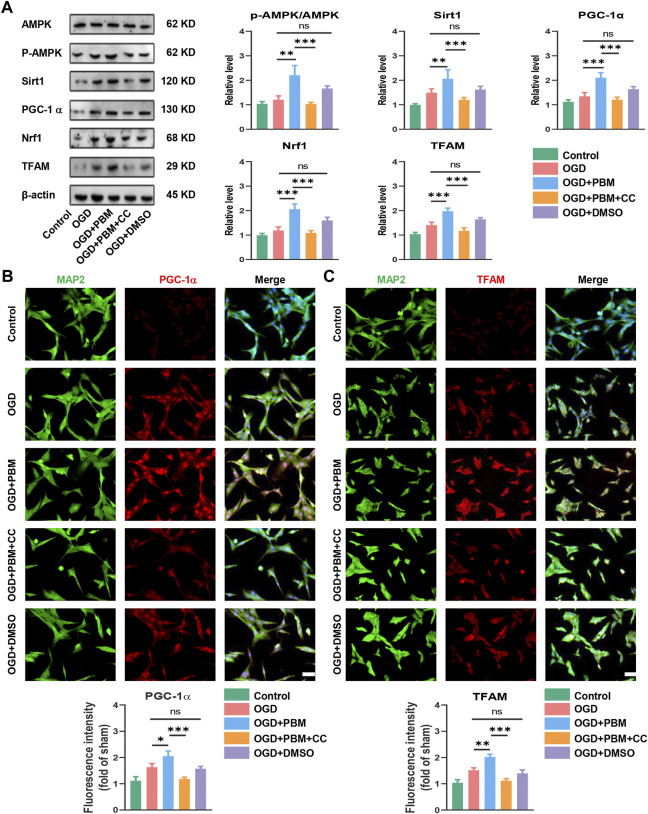
PBM activated AMPK/PGC-1α/TFAM pathway *in vitro*. **(A)**: Western blot analysis and quantification of p-AMPK/AMPK, PGC-1α,TFAM, Sirt1, and Nrf1expression levels in each group, *n* = 4. **(B)**: Representative images of VSC 4.1 (MAP2,green) and PGC-1α (red) for each group. Nuclei were stained with DAPI (blue). Quantification of the intensity of PGC-1α relative to that in the control group, Scale bar: 50 µm, *n* = 3. **(C)**: Representative images of VSC 4.1 (MAP2,green) and TFAM (red) for each group. Nuclei were stained with DAPI (blue). Quantification of the intensity of TFAM relative to that in the control group, Scale bar: 50 µm *n* = 3.

The results noted above suggested that the mechanism of PBM may be related to activation of the AMPK/PGC-1α/TFAM signaling pathway. To verify this, we designed *in vitro* experiments by using CC and PBM *in vitro* following OGD-induced VSC4.1 cell injury. Application of PBM alone could activate this pathway; however, combined application of PBM and CC significantly inhibited activation of the pathway compared with the OGD + PBM group (Figure 5A). VSC4.1 cells labelled with MAP2 and co-stained with PGC-1α or TFAM showed a similar trend (Figures 5B,C).

### Inhabitation of AMPK/PGC-1α/TFAM pathway reversed the effects of Photobiomodulation on mitochondrial bioenergetics and neuronal apoptosis *in vitro*

**FIGURE 6:**
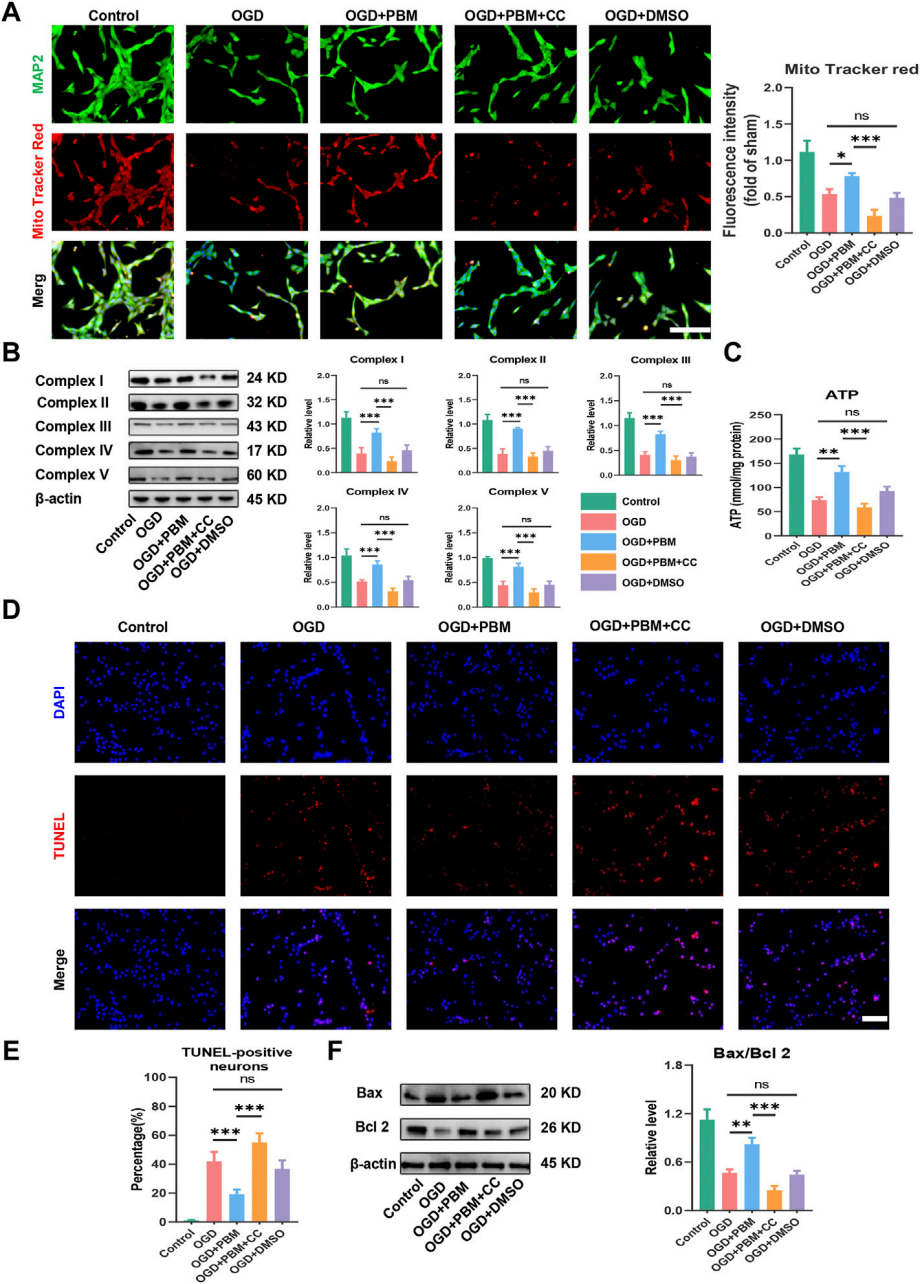
Inhabitation of AMPK/PGC-1α/TFAM pathway reversed the effects of PBM *in vitro*. **(A)**: Representative images of VSC 4.1 (MAP2,green) and Mito Tracker (red) for each group. Nuclei were stained with DAPI (blue). Quantification of the intensity of Mito Tracker red relative to that in the control group, Scale bar: 50 µm *n* = 3. **(B)**: Western blot analysis and quantification of the expression levels of Complex I–V in each group, *n* = 4. **(C)**: Result of ATP production in each group, *n* = 4. **(D,E)**: TUNEL (red) and DAPI (blue) co-stained, Scale bar: 50 µm. The ratio of TUNLE-positive neurons was calculated, *n* = 4. **(F)**: Western blot analysis and quantification of Bax, Bcl2 expression levels in each group, *n* = 4.

Next, we explored the effect of PBM co-treated with CC on mitochondrial function and apoptosis. Mito Tracker Red specifically labels functional mitochondria and we found that PBM intervention could increase the level of functional mitochondria in the OGD model, and this trend was hindered by CC (Figure 6A). Analysis of Complex I–V protein level (Figure 6B) and determination of ATP production (Figure 6C) showed similar trends. Finally, we found that PBM intervention could reduce the level of apoptosis (Figures 6D,E) and reduce the ratio of Bax/Bcl2 after OGD (Figure 6F). Conversely, application of CC terminated the beneficial effects of PBM, contributing to increased expression of Bax, along with a decrease in Bcl-2 expression (Figure 6F).

## Discussion

SCI has a complex pathophysiological process that can be divided into primary and secondary injury. Primary injury is usually irreversible, while secondary injury could be reversible; therefore, the current study is mostly focused on secondary injury. Many processes are involved in secondary injury repair, including calcium signaling, cytoskeletal reorganization, nutrient transportation and antioxidation. All of these processes require mitochondrial bioenergetics (Bradke et al., 2012). Indeed, a decrease in mitochondrial function after SCI hinders functional recovery (Hu et al., 2015).

Therapeutic strategies aimed at regulating mitochondrial bioenergetics have been receiving increasing attention. For example, the glutathione peroxidase simulant Ebselen could improve mitochondrial function by inhibiting oxidative stress and the mitochondria-mediated apoptosis cascade after SCI (Jia et al., 2018). Although some Lithium-containing drugs can enhance mitochondrial respiration in brain and promote neuronal regeneration, they proved invalid in phase II clinical trials (Yick et al., 2004; Maurer et al., 2009; Casha et al., 2012; Yang et al., 2012). Under the premise of systemic administration, it is difficult for drugs to enter the injured area due to the blood-cerebrospinal-fluid barrier (Khadka et al., 2020; Koehn, 2020). Therefore, it is urgent to explore the direct intervention in the injured area.

Our group has developed an implantable optical fiber so that laser bioenergy could directly reach the surface of the spinal cord (Wang et al., 2021a; Wang et al., 2021b; Ma et al., 2022). Compared with percutaneous irradiation, there is no skin or muscle barrier, which improves the efficacy of PBM. The main target (chromophore) for PBM is Complex IV, which is present in the inner membrane of cellular mitochondria as an essential component of the electron transport chain. When photons are absorbed, cytochrome C is stimulated, resulting in an increase in ATP production (Chung et al., 2012). However, the specific pathway through which PBM regulates mitochondrial bioenergetics remains elusive, which limits the clinical application of PBM. Here, we proposed that PBM restored neuronal mitochondrial bioenergetics through the AMPK/PGC-1α/TFAM pathway.

First, we found that application of PBM could promote recovery of motor function and reduce mitochondrial-related neuronal apoptosis (Figure 1). In subsequent studies, we found that PBM activated the AMPK pathway (Figure 2), upregulated expression of p-AMPK, PGC-1α, Nrf1, Sirt1, and TFAM and restored mitochondrial respiratory chain complex activity, leading to increased ATP production (Figure 3). Notably, we observed that motor function recovery and activation of AMPK pathway had a temporal consistency after PBM intervention, which further supports the notion that PBM played a therapeutic role by activating at least one AMPK pathway. In addition, CC could significantly counteract the neuroprotective effect of PBM by inhibiting AMPK pathway activation, thus reducing mitochondrial bioenergetics and promoting neuronal apoptosis (Figure 4). As a next step, we sought to verify the activation of this pathway and any therapeutic effects of PBM *in vitro*. Our *in vitro* experiments showed similar results as *in vivo* experiments (Figures 5, 6).

AMPK is an energy sensor that could be activated by cellular stress, nutrient deficiency, hypoxia, exercise, and drugs to regulate bioenergetics. Overexpression of the AMPKγ3 subunit induces mitochondrial bioenergetics, whereas this effect was absent in AMPK knockout mice (Lee and Kim, 2018). We found expression of p-AMPK increased in the SCI group compared with the Sham group, indicating that the AMPK pathway was activated after injury. While the irreversible structural damage caused by primary injury, the inflammatory reaction and oxidative stress caused by secondary injury are not conducive to cell survival, resulting in a decrease in ATP production. Therefore, interventions are needed to promote ATP production to help minimize and reverse secondary injury. Certain therapeutics, such as Rosmarinus acid, have been suggested to increase mitochondrial bioenergetics by activating the AMPK pathway, but there is still a long way to go before clinical application (Jayanthy et al., 2017).

Sirt1 is a downstream signaling molecule of AMPK that it up regulates PGC-1α through deacetylation. The latter is a key molecule regulating mitochondrial bioenergetics (Cantó and Auwerx, 2009; Zhou et al., 2017). Overexpression of PGC-1α transforms white glycolytic into red oxidative skeletal muscle, accompanied by increased expression of mitochondrial complex II, IV, and Ⅴ mRNA and complex I and IV protein (Lin et al., 2002; Wenz et al., 2009). Knockout of PGC-1α reversed this phenomenon (Arany et al., 2005; Handschin et al., 2007). Our study suggested that upregulation of PGC-1α was accompanied by upregulation of other complex proteins. The difference between our findings and those reported previously may be due to the use of different animal models and treatment measures.

In addition, TFAM, a molecule downstream of PGC-1α, regulates mitochondrial bioenergetics and maintains mitochondrial function by participating in the regulation of mitochondrial DNA transcription and replication (Campbell et al., 2012; Lan et al., 2017). It is worth noting that most mitochondrial proteins are encoded by nuclear genes. Therefore, signals that stimulate mitochondrial bioenergetics need to be transmitted to the nucleus through transcription factors such as Nrf1 (Quirós et al., 2016). Knockout of Nrf1 has been shown to decrease TFAM expression and ATP levels, and to increase the level of apoptotic factors such as Bax, caspase 3, and caspase 9 (Zhang et al., 2017). This is similar to the effect of CC in our study in that CC reduced the expression of p-AMPK, Nrf1, Sirt1, and TFAM, and aggravated neuronal apoptosis. Formoterol, a β2-adrenoceptor agonist, has been approved for treatment of SCI and induces expression of PGC-1α, Nrf1and TFAM (Scholpa et al., 2019b). Recent studies have shown that Formoterol could also reduce skeletal muscle atrophy after SCI (Scholpa et al., 2019a), but it has cardiovascular side effects (Vallorz et al., 2021) so other solutions are needed.

In fact, mitochondrial bioenergetics and apoptosis are two mutually regulated processes. On one hand, insufficient ATP production leads to increased apoptosis. The electron transport chain in the mitochondria is first affected and the integrity of cellular respiration is disrupted after physical factors compress the spinal cord tissue (Scholpa and Schnellmann, 2017). Cell membrane permeability is altered due to lack of energy and initiates the caspase 3-mediated apoptotic cascade pathway through the release of cytokines such as Bax (Jia et al., 2016). Reversing the energy deficit partially reverses apoptosis and promotes axon elongation (Han et al., 2020). On the other hand, reducing apoptosis can also promote ATP production. It was found that Bcl-2, in addition to directly antagonizing Bax, can directly bind to the ATP synthase (complex V) subunit to improve enzyme efficiency and ATP production (Alavian et al., 2011).

Although our study verified that PBM played a neuroprotective role after SCI by improving mitochondrial bioenergetics, it does have limitations that should not be ignored. First, the ideal therapeutic time of PBM is 14 days rather than 28 days. We considered that PBM could rescue partially damaged mitochondria. However, for badly damaged mitochondria, PBM was not effective. Therefore, future studies should consider supplying healthy mitochondria in the injured area and combining with PBM to treat SCI. Second, the use of knockout mice or small interfering RNA would make the experimental design more persuasive. Third, the AMPK pathway also affects mitochondrial bioenergetics by regulating mitochondrial fission and fusion, as well as mitochondrial autophagy (Toyama et al., 2016; Laker et al., 2017). These cellular functions may also play a role in the repair progress and deserve attention in future work.

**FIGURE 7:**
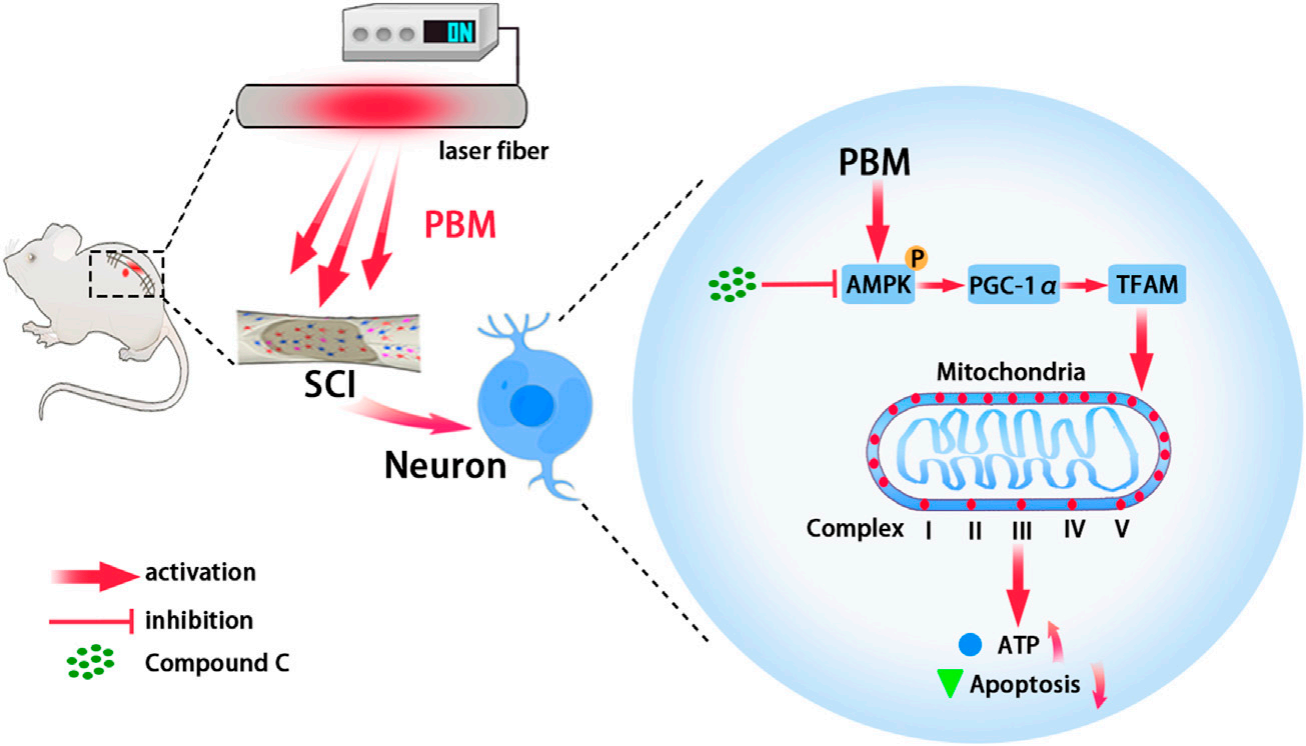
Graphic summary. Embedding fiber mediated PBM restored the activity of mitochondrial respiratory chain complex by activating the AMPK/PGC-1α/TFAM pathway, thus increased ATP production and alleviated apoptosis of neurons after SCI. Picture was exported by FigDraw.

In conclusion, this study showed that PBM played a neuroprotective role by increasing mitochondrial bioenergetics *via* AMPK/PGC-1α/TFAM pathway (Figure 7). These findings suggest that PBM is a potential treatment for SCI.

## Data Availability

The original contributions presented in the study are included in the article/Supplementary Materials, further inquiries can be directed to the corresponding authors.
